# APCSMA: Adaptive Personalized Client-Selection and Model-Aggregation Algorithm for Federated Learning in Edge Computing Scenarios

**DOI:** 10.3390/e26080712

**Published:** 2024-08-21

**Authors:** Xueting Ma, Guorui Ma, Yang Liu, Shuhan Qi

**Affiliations:** 1 School of Computer Science and Technology, Harbin Institute of Technology, Shenzhen 518055, China; 22s151149@stu.hit.edu.cn (X.M.); 22s151091@stu.hit.edu.cn (G.M.); 2 Guangdong Provincial Key Laboratory of Novel Security Intelligence Technologies, Shenzhen 518055, China; 3 Department of Computer Science, Swansea University, Swansea SA1 8EN, UK

**Keywords:** edge computing, federated learning, client selection, model aggregation

## Abstract

With the rapid advancement of the Internet and big data technologies, traditional centralized machine learning methods are challenged when dealing with large-scale datasets. Federated Learning (FL), as an emerging distributed machine learning paradigm, enables multiple clients to collaboratively train a global model while preserving privacy. Edge computing, also recognized as a critical technology for handling massive datasets, has garnered significant attention. However, the heterogeneity of clients in edge computing environments can severely impact the performance of the resultant models. This study introduces an Adaptive Personalized Client-Selection and Model-Aggregation Algorithm, *APCSMA*, aimed at optimizing FL performance in edge computing settings. The algorithm evaluates clients’ contributions by calculating the real-time performance of local models and the cosine similarity between local and global models, and it designs a *ContriFunc* function to quantify each client’s contribution. The server then selects clients and assigns weights during model aggregation based on these contributions. Moreover, the algorithm accommodates personalized needs in local model updates, rather than simply overwriting with the global model. Extensive experiments were conducted on the *FashionMNIST* and *Cifar-10* datasets, simulating three data distributions with parameters dir = 0.1, 0.3, and 0.5. The accuracy improvements achieved were 3.9%, 1.9%, and 1.1% for the *FashionMNIST* dataset, and 31.9%, 8.4%, and 5.4% for the *Cifar-10* dataset, respectively.

## 1. Introduction

With the rapid advancement of science and technology and continuous upgrades in hardware devices, there has been a significant increase in the number of smart terminals and the volume of data they hold. Traditional centralized machine learning training methods, which consolidate data from all clients to the cloud for unified training and optimization, lead to substantial unnecessary communication overhead. This issue becomes even more pronounced in the current 5G/6G network environments, where there are stringent demands for network timeliness, making centralized data processing prone to considerable service latency issues, especially in long-distance transmissions and high-density scenarios. Additionally, increasing concerns over privacy and data security have elevated data leakage risks to a major concern for clients.

Whether due to mistrust towards cloud services or fear of data interception during transmission, there is a growing preference to avoid transferring private data to the cloud. Therefore, in the context of 5G/6G networks, there is a gradual shift of artificial intelligence algorithms towards the edge layer, leading to the emergence of a new deployment model—edge computing [1]. Edge computing enables data processing and analysis at locations closer to the client, significantly reducing service latency and eliminating the need for information exchange between clients and the cloud. This is particularly crucial for real-time or near-real-time application scenarios.

Furthermore, federated learning [2], as a novel distributed learning model specifically designed to solve the problem of data centralization, can jointly train multiple client models without leaking private client data, thereby obtaining a global model. In federated learning, each client has certain computing and storage capabilities and can use local data for local model training. Unlike traditional centralized learning, federated learning clients upload model parameters instead of local data, ensuring the privacy and security of client data through a “data stays, model moves” approach [3]. The server side receives and aggregates these local model parameters uploaded by distributed nodes, calculates and updates the global federated model parameters, then distributes the new model parameters to all client nodes, and repeats the above training process until the model converges or reaches a specified number of training rounds. Since both edge computing and federated learning belong to distributed federated learning and their computation models are similar, the industrial and academic communities have gradually attempted to apply federated learning technology to edge computing scenarios, thus forming edge federated learning [4].

In edge computing scenarios, where client numbers are typically high, edge servers are often unable to aggregate models across all clients for training. To alleviate the computational burden on edge servers and reduce the volume of data transmission from end devices to the edge, traditional federated learning approaches commonly employ random client-selection methods for model aggregation. However, considering the significant heterogeneity of clients in terms of data resources, computational capabilities, and device conditions within the edge computing environment, random selection does not always effectively identify the clients most valuable for global model training. This heterogeneity implies that certain clients may be better suited to participate in specific training rounds, thereby having a more direct impact on enhancing the performance of the global model.

Therefore, we propose the development of a more refined client-selection strategy that can identify and select the clients most likely to improve global model performance in each training round. This strategy will be based on real-time performance indicators of clients, such as data quality, computational capacity, and device stability, ensuring that the chosen clients can maximize their contribution to the optimization and advancement of the global model. By employing this method, we can not only enhance the training efficiency and performance of the model but also utilize edge computing resources more judiciously, paving the way for new possibilities in the application of federated learning within edge computing environments.

Moreover, traditional federated learning typically assigns weights to clients based on the volume of their data. However, since it cannot be guaranteed that all data from each client are effective, it is possible that clients with a larger volume of data may not contribute to the global model training. Therefore, exploring the contribution of clients to the global model training through some real-time dynamic indicators is very necessary. Assigning larger weight parameters to clients with greater contributions can maximize the impact on all clients.

Furthermore, traditional federated learning models usually directly use the global model parameters received by clients to overwrite local models during local updates. This approach can lead to the loss of some client-specific features, which is not conducive to judgments on categories with smaller distribution ranges. Therefore, researching an effective method to balance the relationship between the global model and client local models can, to some extent, preserve some local features of clients, thereby improving the accuracy of the federated model.

The research in this paper focuses on how to solve the federated learning problem in the edge computing scenarios mentioned above, especially for the case of a large number of heterogeneous clients. To this end, we design *APCSMA*, which avoids pre-assessment of the importance of each metric in the current situation by iteratively learning the client’s metric coefficients during the training process, significantly reducing the need for extensive prior experiments. In addition, this approach improves the generalization ability of the model to adapt to different scenarios and datasets more effectively. The main contributions of *APCSMA* are as follows:

(1) We analyze the main factors affecting the efficiency of model training and propose a new contribution function *ContiFunc* based on these factors. By iteratively training this function, *APCSMA* can adaptively determine the coefficients of each metric and evaluate the contribution of clients in real time. This enables us to select appropriate clients to participate in each round of global model training and assign appropriate weights to the selected clients during global model aggregation.

(2) Considering that the number of clients participating in the model-aggregation phase is relatively small and the client contribution values will be used as aggregation weights, we introduce the concept of cosine similarity. The contribution function in the model-aggregation phase is optimized to ensure that each client receives a more accurate aggregation weight value and achieves a fairer global model aggregation, as long as the computational resources and time conditions allow.

(3) In the client local model update phase, we take a different approach from traditional federated learning. By comparing the performance of the aggregated global model with the previous round of local models on client local data, we decide whether to retain certain features of the client local model parameters to achieve personalized model updating.

(4) To verify the feasibility, effectiveness, and generalizability of the proposed method, we conducted extensive simulation experiments and comparative studies using the *FashionMNIST* and *Cifar-10* datasets. By adjusting the Dirichlet coefficient of the dataset distribution, we simulated the degree of data dispersion across various client scenarios. Through comparisons with three classical algorithms—*FedAvg*, *FedPHP* [5], and *FedALA* [6]—the experimental results demonstrate that the *APCSMA* algorithm can adaptively optimize the performance of federated training models in diverse edge scenarios.

The remainder of the paper is organized as follows. Section 2 presents the research background and related literature on edge computing and federated learning. In Section 3, we describe the implementation of traditional federated learning models and the model architecture between terminals and edge nodes in edge scenarios and some influencing factors of model training. Section 4 elaborates on the specific design scheme of the algorithm *APCSMA* proposed in this paper. Section 5 presents extensive validation experiments conducted on two distinct datasets, and includes a comparative analysis with two state-of-the-art algorithms. Finally, we conclude and consider possible future work in Section 6.

## 2. Background and Related Work

Early distributed systems were predominantly based on cloud computing architectures, consisting of a cloud server and multiple clients [7]. In such architectures, servers would collect local data from all clients, upload it to the server, and perform unified parameter aggregation and model updates. However, with the rapid development of Internet of Things technology, the problems faced by this centralized data-processing approach have gradually become apparent: (1) Centralized cloud computing architectures cannot cope with the explosive growth of massive data from clients; (2) Long-distance data transmission between the cloud and edge incurs substantial transportation costs and network latency; (3) There is a risk of leakage of client privacy information during the data-transmission process at the edge. As a result, the network model of edge computing [8] has emerged.

The edge computing architecture improves upon traditional cloud computing by dividing clients into multiple edge network layers and delegating some of the cloud’s computational power to edge network layers near the clients, equipping each edge network layer with edge servers capable of computing and storage. This not only reduces the cost and latency issues associated with long-distance communication between “cloud-edge” but also minimizes the risk of privacy data leakage during long-distance transmission.

Arranging for all clients to participate in the training of the global model is a prerequisite for implementing edge computing and is key to ensuring high-performance services, which is very important for the edge computing planning of enterprises and service providers. Compared to direct long-distance communication between “cloud-terminal”, edge computing introduces an intermediate network layer between the two, namely the edge, dividing all clients into different edge networks.

Federated learning is also a popular distributed training network, first introduced by McMahan et al. [9] and described the most traditional federated algorithm, the Federated Averaging algorithm (*FedAvg*), in previous study [10]. This algorithm improves the performance and training rate of federated learning models by sharing training models through the transmission of client models instead of local training data.

Subsequently, researchers have explored and optimized federated algorithms in various ways. Previous study [11] took into account the system heterogeneity and statistical heterogeneity of the federated network and proposed the *FedProx* federated framework. This framework limits the gap between client models and the current global model by adding a proximal term to the local loss function, ensuring the convergence of the resulting model. Previous study [12] focused on the issue of data heterogeneity, designing the *FedCos* algorithm by analyzing the gradient changes of models during training. This algorithm introduces cosine similarity to reduce the inconsistency in the direction of client models.Personalized Federated Learning is also a method to enhance model performance. Previous study [13] improves performance by identifying sensitive parts of the model, such as the classification layer, and utilizing plugins to distribute information along with model parameters from the server to various clients. Previous study [14] delves into the architecture, development, and evaluation of universal corporate performance, implementing user-centered artificial intelligence. Meanwhile, previous study [15] introduces the Fair Federated Personalized Graph Neural Network (F2PGNN), a graph-based model that addresses inherent bias issues in recommendation systems across different demographic groups.

Moreover, due to the excessive number of clients in edge scenarios, it is not feasible to aggregate local models from all clients. It is necessary to determine which clients should participate in each round of global model training based on an assessment of their contribution to the global model. Existing evaluation metrics include the shapley value [16], single-round and multi-round reconstruction [17], and the differences between the predictions of new and original models [18]. Previous study [19] proposed that selecting clients with larger values of the local loss function could accelerate the convergence of the global model. Previous study [20] simulated the test accuracy of local models using the amount of training data from clients to measure their contribution level.

Since the distributed concepts of edge computing and federated learning are very similar, Previous study [21] introduced an edge server layer in cloud computing and designed a three-layer federated learning algorithm, *HierFAVG*, for aggregating models trained by multiple edge servers. Previous studies [22,23,24,25,26,27,28] focused on the training of clients by the edge and the aggregation of the edge by the cloud, while studies [29,30,31,32,33,34] focused on the terminal-edge, deploying and optimizing in the edge network layer near the clients. Study [29] compares and analyzes feature-selection algorithms from the perspective of accuracy, processing update weights from appropriate users in edge devices. Study [30] designs a mobile-aware collaborative caching approach using asynchronous federated and deep reinforcement learning to cache content at edge or vehicular nodes, thus enabling vehicular computing in edge scenarios. Meanwhile, study [31] employs a self-determination mechanism in place of a centralized selection process, allowing clients to autonomously decide whether to participate in federated training based on resource status, thereby facilitating mobile edge computing.

In summary, recent research has generated significant interest in federated learning and edge computing, particularly regarding their potential for data privacy protection and reducing data-transmission needs. *FedProx* introduces a mechanism to address system heterogeneity, while *FedCos* tackles data heterogeneity by considering the cosine similarity of model gradients. Additionally, personalized federated learning strategies have emerged as a growing trend, aimed at enhancing the applicability of traditional models in various user environments. Despite these contributions establishing a theoretical foundation for federated learning, challenges remain in effectively optimizing model aggregation in environments characterized by system and statistical heterogeneity. Specifically, the critical issues of selecting appropriate clients for each round of model updates and achieving efficient model training under heterogeneous conditions are still unresolved. This research aims to address these challenges by proposing a new solution that combines the latest algorithms and techniques to enhance the application of federated learning in edge computing.

## 3. Adaptive Personalized Client-Selection Algorithm for Edge Federated Learning

The first subsection of this section discusses the challenges faced by traditional federated learning algorithms in edge computing scenarios, particularly the issue of high transmission and computation costs due to the large number of clients. It then emphasizes the limitation of randomly selecting clients, which is not suitable for the heterogeneous nature of clients in edge scenarios, thus affecting the accuracy of the global model. Next, the proposed optimization algorithm is introduced in detail. The drawbacks of the “edge-cloud” computing model are then explained, leading to the proposal of the “terminal-edge-cloud” architecture, along with an introduction to its advantages. In the second subsection, it points out the drawbacks of some federated algorithms that only consider a single metric and provides a detailed introduction to the performance metrics that will be used in this paper’s algorithm.

### 3.1. An Edge Computing Framework Based on “Terminal-Edge” Architecture

In traditional federated learning algorithms, each round of global model training requires the participation of all clients, and it is assumed that the cloud server has sufficient computational power to process the model parameters uploaded by all clients. This poses a challenge in edge computing scenarios, where the multitude of clients means that aggregating all local models to the cloud can lead to significant transmission and computational costs. Due to the differences in data resources, computational, and communication capabilities among clients, randomly selecting a subset of clients for training can reduce the accuracy of the global model. To address these issues, we propose an Adaptive Personalized Client-Selection and Model-Aggregation Algorithm (*APCSMA*) suitable for edge scenarios.

Taking into account the computational capabilities and resource constraints of various clients, to make model training more efficient and not limited by the computational power of individual devices, this paper opts for a streamlined Convolutional Neural Network (*CNN*) structure as the base model for all clients. This choice ensures that even clients with lower computational capabilities can participate in the federated learning process, thus achieving comprehensive client coverage and ensuring the universality and inclusiveness of model training. This not only considers the diversity of computational power distribution but also helps to reduce the communication costs of model training, making the entire federated learning process more feasible and efficient in edge computing scenarios.

Building on this, our approach involves delegating some of the cloud’s computational capabilities to the edge layer closer to the clients, to alleviate the load on the central server and reduce communication latency. Specifically, we divide the clients into different edge network layers and equip each layer with an edge server capable of computing and storage. These edge servers are responsible not only for aggregating the global model but also for saving the performance metrics of all clients from the last participation in global training. By conducting small-scale training between edge servers and clients within each edge network layer, our architecture can effectively optimize the terminal-to-edge model training process. This “terminal-edge-cloud” architecture not only reduces communication overhead and improves the efficiency of model updates aggregation but also ensures data privacy throughout the learning process.

**Figure 1:**
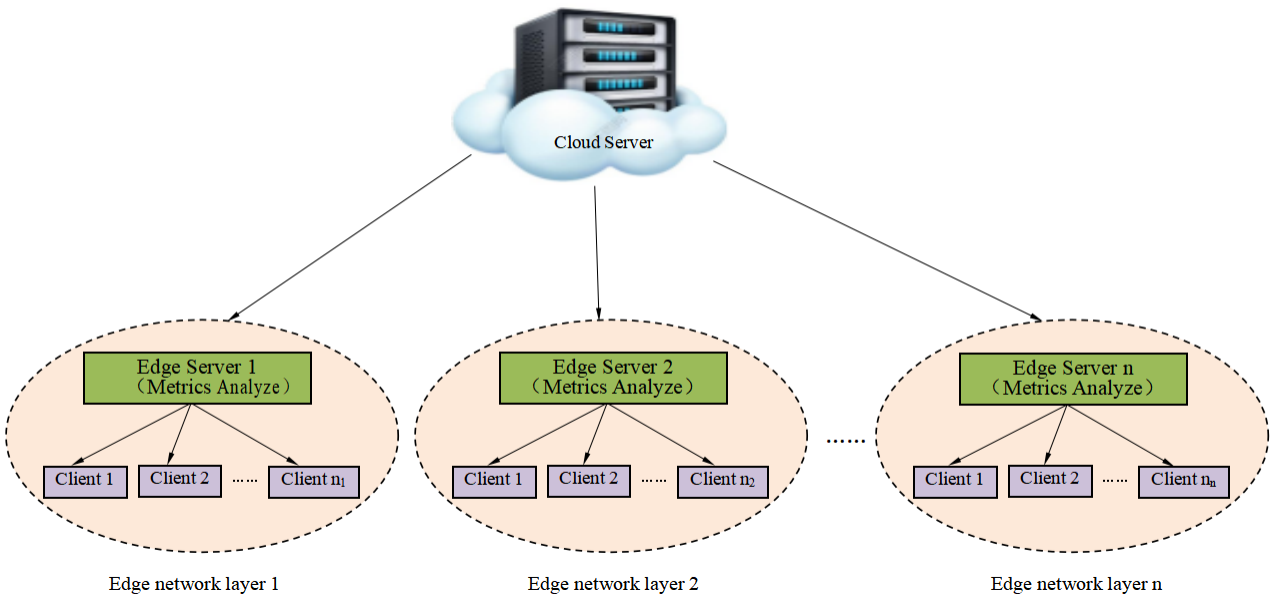
“Terminal-edge-cloud” architecture in edge computing (incorporating multiple edge layers).

Furthermore, we have implemented various algorithms to address the heterogeneity of data distribution, ensuring the generalizability of the model and its robustness in *Non-IID* data environments. Through this horizontal federated learning architecture, our research demonstrates a collaborative machine learning method that can effectively utilize data resources scattered among various data holders while maximally protecting data privacy. The entire “terminal-edge-cloud” federated learning framework is illustrated in Figure 1, with the main optimization being the training conducted within each edge network layer, that is, the optimization of the model training between the terminals and the edge.

This design effectively mitigates the challenges of federated learning in edge computing scenarios. By decentralizing computational capabilities to the edge layer, it not only safeguards the privacy of client data to a certain extent but also alleviates the computational burden on the cloud and reduces transportation costs, thereby enhancing the efficiency of model training. Moreover, through small-scale training within edge network layers and analysis of metrics across various edge repositories, it better accommodates the differences among clients, thereby improving the performance of the global model. This adaptive federated learning algorithm offers a viable solution for edge computing and holds broad application prospects.

### 3.2. Factors Influencing Client Selection and Weight Allocation

Given the limited number of clients that can participate in global aggregation in each round, selecting the appropriate clients is crucial. The most common method of client selection is the random selection algorithm, an unbiased selection method. While ensuring that each client has an equal probability of participation in the global model training process, the heterogeneity of clients in terms of data and computational resources means that a random selection algorithm is unable to identify the clients most beneficial for enhancing global performance in each training round. Therefore, researchers have attempted to study biased client-selection strategies.

In existing research, server-side selection of clients has been based on metrics such as the number of local training samples available at the client [10] and the loss function [19]. However, these metrics focus on relatively narrow aspects and do not adequately measure each client’s contribution to the optimization of global model performance during aggregation. Therefore, this paper considers multiple client metrics within model training and employs a function design approach to interrelate these metrics. Among these, the most common metrics for classification tasks include the following:

(1) Client sample number (number): The volume of test sample data in each client’s local data.

(2) Participation rounds (epoch): The total number of rounds each client participates in global training.

(3) Accuracy (acc): Accuracy is the most direct evaluation indicator in classification tasks, i.e., the proportion of correctly predicted samples out of the total number of samples. Whether a classification is correct or not only considers whether the predicted category matches the true category, as shown in Equation (1).

(1) $$acc=\frac{TP+TN}{TP+TN+FP+FN}$$

where, TP, TN, FP, and FN stand for True Positive, True Negative, False Positive, and False Negative, respectively.

(4) Loss Function (loss): The cross-entropy loss function is most commonly used in classification problems to calculate the discrepancy between model predictions and the true labels of the samples, thereby reflecting the performance of the model in federated training. In the case of binary classification problems, the cross-entropy loss for an individual sample is denoted as loss, while the cross-entropy loss for a client with *N* samples is denoted as *L*. The calculation methods for these are shown in Equations (2) and (3), respectively (for multi-class problems, the cross-entropy loss function is typically combined with the softmax function to extend it to the multi-class cross-entropy loss function).

(2) $$loss=-\left(ylog\hat{y}+\left(1-y\right)log\left(1-\hat{y}\right)\right)$$

(3) $$L=\sum_{i=1}^{N}loss=-\sum_{i=1}^{N}\left(y_{i}log\hat{y_{i}}+\left(1-y_{i}\right)log\left(1-\hat{y_{i}}\right)\right)$$

where, *N* represents the number of test samples at each client, *i* denotes the *i*-th training sample of the client, *y* and $\hat{y}$ respectively represent the actual label and the predicted label of the *i*-th sample.

## 4. Algorithm

This section is the most critical part of the entire text, providing a detailed overview of the three optimization steps discussed in this paper. In the first subsection, we introduce the contribution function, offering an in-depth explanation, followed by a description of its specific applications during the client-selection phase and the model-aggregation phase, along with flowcharts and pseudocode. Recognizing that the model-aggregation phase demands more detailed considerations than the client-selection phase, the second subsection introduces the concept of cosine similarity. We then explain how to appropriately incorporate cosine similarity in the experiments and the subsequent optimization methods. Finally, the third subsection addresses the traditional local model updating methods in federated learning, highlights their shortcomings, and proposes a conditional weighted update approach for the client-local models.

### 4.1. Client Selection and Model Aggregation

While the number of client samples, the number of participation rounds, the accuracy of the local model, and the loss function all influence the training of the global model, balancing the impact of different metrics on the training of the global model is a crucial and complex process. Therefore, we have established a contribution function, *ContriFunc*, as shown in Equation (4).

(4) $$ContriFunc=\alpha \cdot Num_{C_{i}}+\beta \cdot Epoch_{C_{i}}+\gamma \cdot Acc_{C_{i}}+\eta \cdot Loss_{C_{i}}$$

where, *i* denotes the ID number of each client, $C_{i}$ represents the *i*-th client, and α, β, γ, and η correspond to the relative importance coefficients of the number of samples, the number of participation rounds, the accuracy, and the loss function value of the contribution function ContriFunc of each client, respectively, with the constraint that α+β+γ+η=1.

The determination of the metric coefficients within the contribution function typically relies on existing empirical knowledge or tedious experimental processes. Once these coefficients are set up, this algorithm can be only applicable to specific datasets in a particular environment, showing strong context dependencies. Such an approach not only consumes a considerable amount of time and effort but also has relatively narrow applicability. In practical applications, the need for frequent recalculations significantly adds to the inconvenience of operations, thereby affecting the practicality of the method.

To address the aforementioned issues, this paper proposes an adaptive method for calculating metric coefficients. This method employs a simple neural network model that automatically learns and determines the weights of each metric in the contribution function through an iterative training process. The network model is capable of automatically adjusting the metric coefficients based on real-time performance feedback, significantly reducing reliance on traditional manual parameter tuning. Moreover, the model exhibits good adaptability and can efficiently process a variety of datasets in different scenarios.

To effectively train the aforementioned neural network, this paper constructs a shared dataset, $D_{share}$. This dataset consists of data fragments provided by various clients, intended to collect diverse data characteristics while retaining the uniqueness of each client’s data. This approach ensures that the coefficient training dataset not only covers a wide range of heterogeneous data types but also integrates the data variability between different clients, providing a solid foundation for the comprehensive training of the model.

The contribution function ContriFunc proposed in this paper is primarily applied in two stages. First, during the client-selection phase, the server-side inputs the performance metrics of each client’s local model into the contribution function to autonomously and in real-time calculate the contribution level of all clients to the global model. It then filters out a subset of clients that are deemed to have a higher contribution to the training of the global model for that round. These selected clients will participate in the training of the global model for the current round. Subsequently, during the client model-aggregation phase, the server-side uses the function to accurately compute the contribution of these selected clients to the global model. Based on the magnitude of their contributions, the server then more justly and efficiently allocates the aggregation weights. This method not only optimizes the model training process but also enhances the overall performance and fairness of the model.

#### 4.1.1. Adaptive Client Selection Based on Contribution Function

In the client-selection phase, each client uploads the performance indicators of their local model from the previous training round to the edge server. The server initially uses iterative training on a shared dataset to derive coefficients for the indicators that are appropriate for the client-selection stage. Subsequently, it incorporates each client’s indicators into the contribution function to calculate the contribution value for all clients. These values are used to assess each client’s contribution to the training of the global model, and a subset of clients with higher contributions are selected to participate in the current round of global model training.

To illustrate the adopted method, this paper takes the accuracy of the client’s local model as an example. First, an accuracy vector all_train_acc is defined, which contains the model accuracy of all clients from the previous training round. Then, the ratio of each client model’s accuracy to the total accuracy of all models is calculated, thereby generating a normalized accuracy distribution vector ls_acc. This normalized vector reflects the relative importance of each client model’s accuracy within the accuracy of all client models. The normalization process is shown in Equation (5).

(5) $$ls\text{\_}acc=\frac{all\text{\_}train\text{\_}acc}{\sum_{i=1}^{m}\left({all\text{\_}train\text{\_}acc}_{i}\right)}$$

where, *m* represents the number of clients, ls_acc is the normalized distribution vector of all clients’ accuracies, and ${all\text{\_}train\text{\_}acc}_{i}$ represents the local model accuracy of client i.

To ensure mathematical certainty in subsequent analyses, such as avoiding the base of a logarithm being zero when performing log transformations, this study has made a minor positive adjustment to each element of the normalized accuracy distribution vector ls_acc. Specifically, a very small positive number ϵ is added to each element of the vector to ensure that the value of each element is greater than zero when performing logarithmic operations or other computations that could lead to uncertainty. The process of this positive adjustment is shown in Equation (6).

(6) $$ls\text{\_}acc=\left\{\begin{array}{cc} 1\times 10^{-6} & ls\text{\_}acc=0,\\ ls\text{\_}acc & \mathrm{otherwise}. \end{array}\right.$$

where ls_acc is the normalized distribution vector for all client precision and this formula represents batch operations on the elements in the vector.

Subsequently, to quantify the amount of information contained in the accuracy of each client’s model, the concept of information entropy is introduced in this study. Specifically, by applying a negative logarithmic transformation to the normalized accuracy distribution vector ls_acc, the information entropy of each client’s local model within the accuracy distribution is calculated. The calculation process of the information entropy is shown in Equation (7).

(7) $$ls\text{\_}acc\text{\_}inf=-\log_{2}\left(ls\text{\_}acc\right)$$

To convert the calculated information quantity vector into a probability distribution, the study normalizes this vector to ensure that the sum of all elements in the vector equals 1, thus meeting the basic requirements of a probability distribution. The normalized information quantity vector more reasonably reflects the relative contribution of information from each client’s model to the overall system. The corresponding normalization process is shown in Equation (8).

(8) $$W\text{\_}acc=\frac{ls\text{\_}acc\text{\_}inf}{\sum_{i=1}^{m}\left({ls\text{\_}acc\text{\_}inf}_{i}\right)}$$

In this study, the local model accuracy, the number of training rounds participated, and the local model loss function values of the client all need to go through the aforementioned logarithmic normalization process to ensure that these indicators are numerically stable and reflect the actual amount of information when applied in the contribution function. However, the normalization method for the client’s data volume is slightly different. The ratio of the local data volume of the client to the total data volume of all clients is used directly as the data volume indicator for each client in the contribution function. The calculation formula for this indicator is shown in Equation (9).

(9) $$W\text{\_}num=\frac{\mathrm{num}}{\sum_{i=1}^{m}\left({\mathrm{num}}_{i}\right)}$$

The contribution function comprehensively considers the normalized values of various indicators from clients to evaluate the contribution of each client to the global model training. This evaluation serves as the basis for decision-making in the client-selection phase, determining which clients are chosen to participate. The formula for this function is shown in Equation (10).

(10) $$ContriFunc_{i}=\alpha_{1}\cdot W_{i}\text{\_}num+\beta_{1}\cdot W_{i}\text{\_}epoch+\gamma_{1}\cdot W_{i}\text{\_}acc+\eta_{1}\cdot W_{i}\text{\_}loss$$

where, $\alpha_{1}$, $\beta_{1}$, $\gamma_{1}$, and $\eta_{1}$ represent the respective coefficients for each metric of a client during the client-selection phase within the contribution function. The variable *i* denotes the client’s ID number. The terms $W_{i}\text{\_}num$, $W_{i}\text{\_}epoch$, $W_{i}\text{\_}acc$, and $W_{i}\text{\_}loss$ correspond to the normalized values of data volume, number of epochs participated, model accuracy, and model loss, respectively, for the local model of client *i* in the previous round of the global model.

To facilitate a more convenient and accurate reflection of the relative importance of each metric within the contribution function, this study employs an adaptive iterative optimization method to determine the corresponding coefficients for each metric. This method utilizes a simple neural network trained on a shared dataset. Through the iterative training of this simple neural network, the most suitable coefficients for each metric in each round are derived. The process of iterative training is illustrated by Equation (11).

(11) $$\alpha_{1}^{*},\beta_{1}^{*},\gamma_{1}^{*},\eta_{1}^{*}=\arg\min_{\alpha_{1},\beta_{1},\gamma_{1},\eta_{1}}Loss_{share}\left(\sum_{i=1}^{m}ContriFunc_{i}\cdot W_{i}^{t};D_{share}\right)$$

where, *m* represents the number of clients within the edge computing scenario. $W_{i}^{t}$ and $ContriFunc_{i}$ denote the local model parameters for client *i* from the previous round of global training and the corresponding contribution function values, respectively. $D_{share}$ is a specially constructed shared dataset featuring characteristics from all clients, which is used for the iterative training of the simplified model.

By incorporating a contribution function during the client-selection phase, we can evaluate the extent of each client’s contribution to the current round of global training based on the values of their respective performance metrics and corresponding coefficients. Clients are ranked in descending order of their contribution levels, and a subset of clients with higher contribution levels is selected to participate in the global model training.

**Figure 2:**
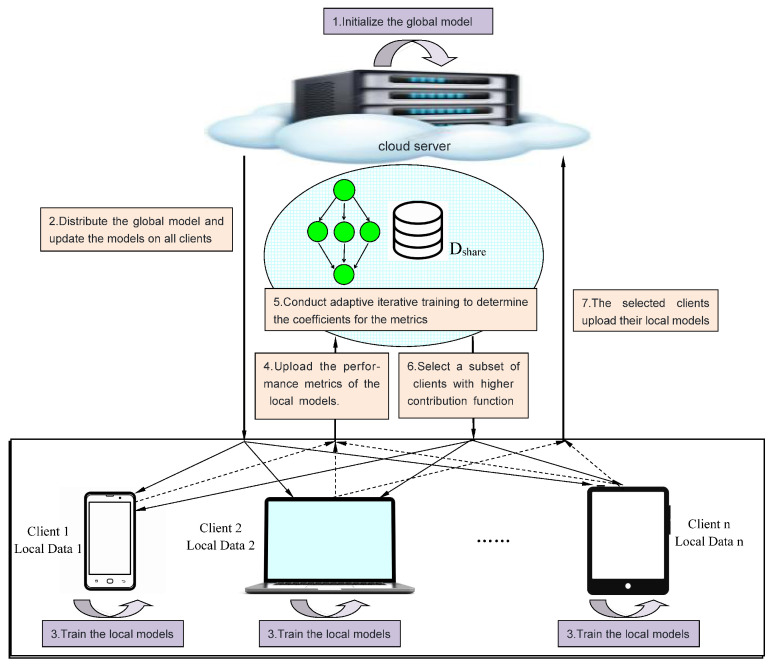
Adaptive client selection based on contribution function values for global federated training.

This approach enhances the utilization of edge computing and federated learning benefits, thereby improving the performance metrics of the global model. The overall process of client selection and the corresponding pseudocode are depicted in Figure 2 and Algorithm 1, respectively.
**Algorithm 1** Client selection with self-learning weight optimization**Require:** number of clients nc, proportion of selected clients jr, shared dataset D_share, metric values for each client num, epoch, acc, and loss. **Ensure:** List of selected clients participating in each round of global training select_clients. 1: **procedure**
ChooseClients 2:     get acc,loss,cnt,num from all clients 3:     W_acc← log normalization(acc) 4:     W_loss← log normalization(loss) 5:     W_cnt← log normalization(cnt) 6:     W_num←Normalize(num) 7:     $(\alpha_{1},\beta_{1},\gamma_{1},\eta_{1})\leftarrow$ Call F1Parameters 8:     **for** i←0 to nc **do** 9:         $ContriFunc_{i}\leftarrow \alpha_{1}\cdot W_{i}\text{\_}num+\beta_{1}\cdot W_{i}\text{\_}epoch+\gamma_{1}\cdot W_{i}\text{\_}acc+\eta_{1}\cdot W_{i}\text{\_}loss$  10:     **end for**  11:     select_clients←ContriFunc[:nc∗jr]  12:     **return** select_clients  13: **end procedure**  14: **function**
F1Parameters  15:     Initialize model, optimizer, and loss function  16:     Prepare D_share and set parameters of model  17:     $\alpha_{1},\beta_{1},\gamma_{1},\eta_{1}\leftarrow$ 1/4, 1/4, 1/4, 1/4  18:     **for** each training epoch **do**  19:         $\left(\alpha ,\beta ,\gamma ,\eta \right)\leftarrow \min L(Func,W^{t};D_{share})$  20:     **end for**  21:     $(\alpha_{1}^{*},\beta_{1}^{*},\gamma_{1}^{*},\eta_{1}^{*})\leftarrow$ coefficients of last round  22:     **return** $\left(\alpha_{1}^{*},\beta_{1}^{*},\gamma_{1}^{*},\eta_{1}^{*}\right)$  23: **end function**

#### 4.1.2. Adaptive Aggregation of Client Models Based on Contribution Function

In the global model-aggregation phase, the server is also required to collect various indicators submitted by clients and perform normalization. Additionally, it adaptively derives the coefficients corresponding to each indicator in the contribution function through iterative training on the shared dataset. The specific methods for indicator normalization and the determination of contribution function coefficients are consistent with those in the client-selection phase. The contribution function for the aggregation phase is shown in Equation (12).

(12) $$ContriFunc_{i}=\alpha_{2}\cdot W_{i}\text{\_}num+\beta_{2}\cdot W_{i}\text{\_}epoch+\gamma_{2}\cdot W_{i}\text{\_}acc+\eta_{2}\cdot W_{i}\text{\_}loss$$

where, $\alpha_{2}$, $\beta_{2}$, $\gamma_{2}$, and $\eta_{2}$ represent the corresponding coefficients of various indicators for all selected clients during the model-aggregation phase in the contribution function, respectively. The variable *i* denotes the ID number of a client, $W_{i}\text{\_}num$, $W_{i}\text{\_}epoch$, $W_{i}\text{\_}acc$, and $W_{i}\text{\_}loss$ represent the normalized values of the indicators for the local model of the selected client *i* from the previous round of global training.

Unlike the client-selection phase, the aggregation phase does not need to consider all clients in the edge scenario but only focuses on those who have been selected to participate in the current round of global training. During this phase, the outcome of the contribution function not only reflects the contribution level of each selected client to the current round of global training but also determines their participation weight in the global model training process. In other words, the contribution function result for each selected client will serve as the fusion ratio for integrating each client’s local model in the weighted aggregation process of the global model. The process during the model-aggregation phase is shown in Equation (13).

(13) $$W^{t+1}=\sum_{k=1}^{K}\left(ContriFunc_{k}W_{k}^{t}\right)$$

where, *K* denotes the number of clients participating in the training of the global model per round, and *K* identifies the *k*-th selected client. The notations t+1 and *t* correspond to the current and previous rounds of global model training, respectively. The terms $w_{k}^{t}$ and $ContriFunc_{k}$ represent the local model gradient of the *k*-th selected client from the previous round and the weight coefficient calculated by the contribution function for this client in the aggregation phase of the global model for the current round, respectively. $W^{t}$ and $W^{t+1}$ indicate the global model gradients before and after aggregation, respectively.

**Figure 3:**
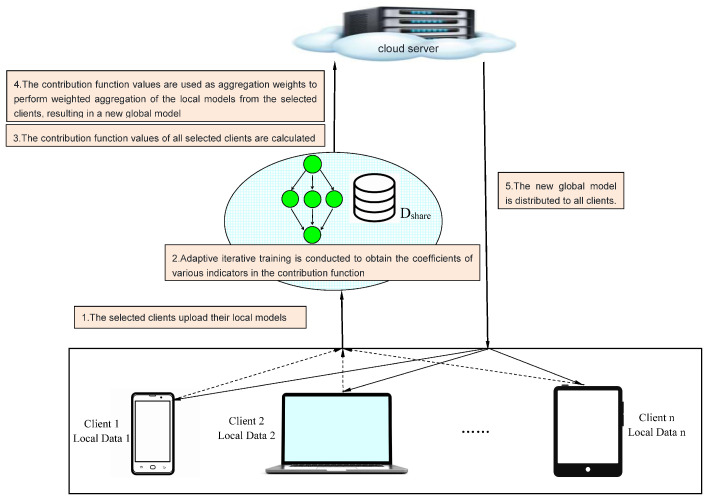
Adaptive aggregation of the model gradients from the selected clients based on the values of the contribution function.

By introducing the contribution function during the global model-aggregation phase, this study achieves a quantitative assessment of the contribution of the local models from the selected clients, thereby enhancing the precision and efficiency of the global model training. The overall process and pseudocode for the client model-aggregation phase are illustrated in Figure 3 and Algorithm 2, respectively.
**Algorithm 2** Model aggregation with self-learning weight optimization**Require:**
Current epoch t+1 and previous epoch *t*, global model parameters before model aggregation $W^{t}$, selected clients select_clients along with local model gradients ${W_{i}}^{t}$ and performance metrics ${W_{i}\text{\_}num}^{t}$, ${W_{i}\text{\_}epoch}^{t}$, ${W_{i}\text{\_}acc}^{t}$ and ${W_{i}\text{\_}loss}^{t}$ in epoch *t*. **Ensure:**
The global model parameters after model aggregation in each round $W^{t+1}$. 1: **procedure**
ModelAggregation 2:     get acc,loss,cnt,num from selected clients 3:     W_acc← log normalization(acc) 4:     W_loss← log normalization(loss) 5:     W_cnt← log normalization(cnt) 6:     W_num←Normalize(num) 7:     $(\alpha_{2},\beta_{2},\gamma_{2},\eta_{2})\leftarrow$ Call F2Parameters 8:     **for** i←0 to nc∗jr **do** 9:         $ContriFunc_{i}\leftarrow \alpha_{2}\cdot W_{i}\text{\_}num+\beta_{2}\cdot W_{i}\text{\_}epoch+\gamma_{2}\cdot W_{i}\text{\_}acc+\eta_{2}\cdot W_{i}\text{\_}loss$  10:     **end for**  11:     $W^{t+1}\leftarrow \sum_{k=1}^{nc*jr}\left({\mathrm{ContriFunc}}_{k}\cdot W_{k}^{t}\right)$  12:     Send the new global model to all clients  13:     **return** $W^{t+1}$  14: **end procedure**  15: **function**
F2Parameters  16:     Initialize model, optimizer, and loss function  17:     Prepare D_share and set parameters of model  18:     $\alpha_{2},\beta_{2},\gamma_{2},\eta_{2}\leftarrow 1/4,1/4,1/4,1/4$  19:     **for** each training epoch **do**  20:         $\left(\alpha ,\beta ,\gamma ,\eta \right)\leftarrow \min L(Func,W^{t};D_{share})$  21:     **end for**  22:     $(\alpha_{2}^{*},\beta_{2}^{*},\gamma_{2}^{*},\eta_{2}^{*})\leftarrow$ coefficient of last round  23:     **return** $\left(\alpha_{2}^{*},\beta_{2}^{*},\gamma_{2}^{*},\eta_{2}^{*}\right)$  24: **end function**


### 4.2. Optimization of Model Aggregation Based on Cosine Similarity

This section focuses on optimizing the contribution function during the global model-aggregation phase. Unlike the client-selection phase, where clients are chosen solely based on the ranking of their contribution function values, in this phase, the contribution function of the selected clients serves as their weight during model aggregation. Consequently, more precise calculations are required. Additionally, only clients chosen to participate in the current round of global model aggregation will compute their contribution functions, significantly reducing computational costs and pressure. This allows us to introduce additional metrics for more nuanced calculations, specifically incorporating the metric of cosine similarity.

#### 4.2.1. Cosine Similarity between Neural Network Models

In this study, we also introduce the concept of cosine similarity, a similarity measurement method widely applied in fields such as text and image analysis and processing. Within the context of federated learning, cosine similarity serves as a crucial metric for evaluating the extent of client model updates, quantifying the similarity between each client model and the global model. This concept is incorporated into the design of the contribution function during the model-aggregation phase, allowing for a more precise assessment of the contribution level of the selected clients’ local models during aggregation. The traditional method of calculating cosine similarity evaluates the similarity between different vectors by measuring the cosine of the angle between two vectors, a result that is independent of the vectors’ magnitudes and solely related to their directions. The cosine similarity between two vectors can be represented by cos(θ), and the calculation method is shown in Equation (14):

(14) $$cos\theta =\frac{w_{1}\cdot w_{2}}{∥w_{1}∥∥w_{2}∥}$$

where, $w_{1}$ and $w_{2}$ denote two vectors, while θ represents the angle between them, and $|w_{1}|$ and $|w_{2}|$ correspond to the magnitudes of the vectors, respectively.

However, within the training process of federated learning, the cosine similarity to be computed is not between two simple vectors but rather between two convolutional neural network (CNN) models. The advantage of employing cosine similarity is its insensitivity to the dimensionality of vectors, rendering it appropriate for the computation of similarity between parameters of diverse CNN models. Convolutional neural networks are comprised of multiple layers, with each layer’s parameters being representable as a vector. Considering the data heterogeneity and category-distribution variances among clients in different edge computing scenarios, the cosine similarity between parameter vectors of each layer in the local models of clients and the global model on the server side varies accordingly. Consequently, one may first convert the parameter vectors of both the local and global models into a uniform one-dimensional vector format, thereby facilitating the assessment of similarity between the models of clients and the server. A higher cosine similarity value relative to the global model signifies a closer alignment of the client’s local model gradient direction with that of the global model, indicating lesser personalization, and vice versa.

#### 4.2.2. Model Aggregation with Weight Function Modification

In this study, we employ a segregated neural network architecture, BaseHeadSplit, which is composed of two parts: the base network and the head network. The base network is a convolutional neural network named *FedAvgCNN*, which includes two convolutional layers and one fully connected layer. Specifically, the first convolutional layer, conv1, consists of a 5×5 convolutional kernel with a single-channel input, producing 32 feature maps, followed by a ReLU activation function and a 2×2 max pooling layer. The second convolutional layer, conv2, expands the features to 64 and similarly utilizes a ReLU activation function and max pooling layer. Subsequently, the fc1 layer maps the 1024 features outputted by the convolutional layers to 512 features through a fully connected layer, followed by activation with the ReLU function. The final layer of the base network, FC, is set to the Identity function, allowing the features to be passed to the head network without altering their representation. The head network consists of a single-layer fully connected network, whose primary function is to map the features extracted by the base network to 10 output classes, suitable for classification tasks.

To assess the similarity between the global model and the local models of selected clients during the federated learning process, we calculate the cosine similarity of each local model to the global model separately for the base network and the head network components. By applying logarithmic normalization to these similarity values, we obtain standardized similarity metrics, denoted as W_base and W_head. These metrics are then integrated into the contribution function *ContriFunc*, serving as crucial indicators for adjusting and optimizing the model-aggregation strategy. The optimized contribution function *ContriFunc’*, is presented as shown in Equation (15).

(15) $$\begin{array}{c} ContriFunc_{i}^{\prime }=\alpha_{2}\cdot W_{i}\text{\_}num+\beta_{2}\cdot W_{i}\text{\_}epoch+\gamma_{2}\cdot W_{i}\text{\_}acc+\\ \eta_{2}\cdot W_{i}\text{\_}loss+\mu_{2}\cdot W_{i}\text{\_}base+\nu_{2}\cdot W_{i}\text{\_}head \end{array}$$

where, $W_{i}\text{\_}base$ and $W_{i}\text{\_}head$ represent the log-normalized cosine similarity values between the base and head networks of the selected client *i*’s local model and the global model, respectively. The coefficients $\mu_{2}$ and $\nu_{2}$ correspond to these two metrics and are utilized to adjust the influence of the base and head network similarities in the assessment of contribution.

### 4.3. Conditional Weighted Updating of Client Local Models

After global model aggregation, the server disseminates the updated global model to all clients. In traditional federated learning practices, clients typically overwrite their local models with the new global model upon receipt. However, this approach may lead to a loss of the local model’s adaptation to the client’s unique data distribution. To mitigate this issue, this study proposes an optimization strategy that determines the update policy by comparing the performance of the global model with the local model on the client’s data. Specifically, the client validates its local dataset using both the current local model and the received global model to obtain a local accuracy Acc_local and a global accuracy Acc_global. Based on the comparison of these two accuracies, the client will decide whether to directly overwrite the local model with the global model or integrate the global and local models using a weighted combination. The aforementioned conditional weighted update process is shown in Equation (16).

(16) $$W_{i}^{t+1}=\left\{\begin{array}{cc} W^{t+1} & \mathrm{if}\;Acc_{\mathrm{global}}\geq Acc_{\mathrm{local}},\\ \left(1-\kappa \right)W^{t+1}+\kappa W_{i}^{t} & \mathrm{otherwise}. \end{array}\right.$$

where, $W^{t+1}$ represents the new global model obtained from the current round of model aggregation, $W_{i}^{t}$ and $W_{i}^{t+1}$ respectively denote the local model gradients of client *i* before and after the model update. Acc_global and Acc_local indicate the accuracy of the new global model and the previous local model validated on the local dataset, respectively. The parameter κ within the range [0, 1] is a tuning factor used to control the influence weight of each local mode parameter during the update process. This factor can be dynamically adjusted based on the performance of the local model or set to a fixed value according to a predefined strategy.

This method allows clients to retain a degree of local characteristics during the global model update process, thereby enhancing the adaptability and robustness of the model on specific client data. Moreover, by finely controlling the degree of integration between global model parameters and local model parameters, this approach helps to balance the trade-off between global optimization objectives and the maintenance of client data specificity. The pseudo-code for this procedure is shown in Algorithm 3.
**Algorithm 3** Conditional weighted updating of client local model**Require:** Each local model of previous epoch $W_{i}^{t}$, global model after aggregation of current epoch $W^{\left(t+1\right)}$, conditional weight of each local model κ. **Ensure:** Each updated local model of current epoch $W_{i}^{t+1}$. 1: **procedure**
LocalModelUpdate 2:     Each client receives the updated global model $W^{\left(t+1\right)}$ 3:     **if** $Acc_{global}\geq Acc_{local}$ **then** 4:          $W_{i}^{t+1}\leftarrow W^{\left(t+1\right)}$ 5:     **else** 6:          $W_{i}^{t+1}\leftarrow \left(1-\kappa \right)\times W^{t+1}+\kappa \times W_{i}^{t}$ 7:     **end if** 8:     **return** $W_{i}^{t+1}$ 9: **end procedure**

## 5. Experiment

In this section of our research, we conducted a series of experiments using the *FashionMNIST* and *Cifar-10* datasets. To validate the robustness of our proposed algorithm in adapting to varying degrees of client data distribution, we carried out experiments on these datasets with three different levels of data heterogeneity. Initially, we thoroughly explored each optimization step of the *APCSMA* algorithm and experimentally verified its effectiveness. Subsequently, we plotted the accuracy and loss function over the course of training in line graphs to visually demonstrate the performance dynamics of the algorithm. Finally, we conducted a horizontal comparison of our algorithm with the current leading *FedALA* and *FedPHP* algorithms to fully showcase the efficiency and performance advantages of our proposed algorithm.

### 5.1. Dataset Introduction and Dirichlet Distribution Partitioning

To demonstrate the effectiveness and generalizability of our algorithm, we conducted experiments on the *FashionMNIST* and *Cifar-10* datasets respectively. The *FashionMNIST* dataset consists of 70,000 front-view images of various products across 10 categories, with each category containing 6000 training samples and 1000 test samples. Each image sample is a 28×28 pixel grayscale image of clothing. The *Cifar-10* dataset, widely used for image recognition tasks, comprises 60,000 color images of 32×32 pixels, divided into 10 categories, with each category having 6000 images.

To simulate the data distribution in the real world, we employed the Dirichlet distribution to generate *Non-IID* data partitions. The Dirichlet distribution is a multivariate probability distribution that is shaped by the parameter vector $\alpha =(\alpha_{1},\alpha_{2},\ldots ,\alpha_{K})$, where *K* represents the number of categories. In this experiment, a symmetric Dirichlet distribution was used, where the parameter vector $\alpha_{i}$ has the same value for each category; thus, it can be denoted as α=(α,α,...,α). Given the parameter α, the Dirichlet distribution can produce a corresponding *K*-dimensional vector $p=(p_{1},p_{2},\ldots ,p_{K})$, where $P_{i}$ indicates the proportion of the *i*-th category, satisfying $\sum_{i=1}^{K}P_{i}=1$. The probability density function of this symmetric Dirichlet distribution is shown in Equation (17).

(17) $$Dir\left(P|\alpha \right)=\frac{1}{B(\alpha )}\prod_{k=1}^{K}P_{k}^{\alpha_{k}-1}$$

where, P is a random variable vector that follows a Dirichlet distribution. The parameter vector α is symmetrical for the Dirichlet distribution and controls the shape of the distribution. B(α) is the multivariate β function, which ensures the normalization of the probability density function. *K* denotes the dimensionality of the random variable P.

**Figure 4:**
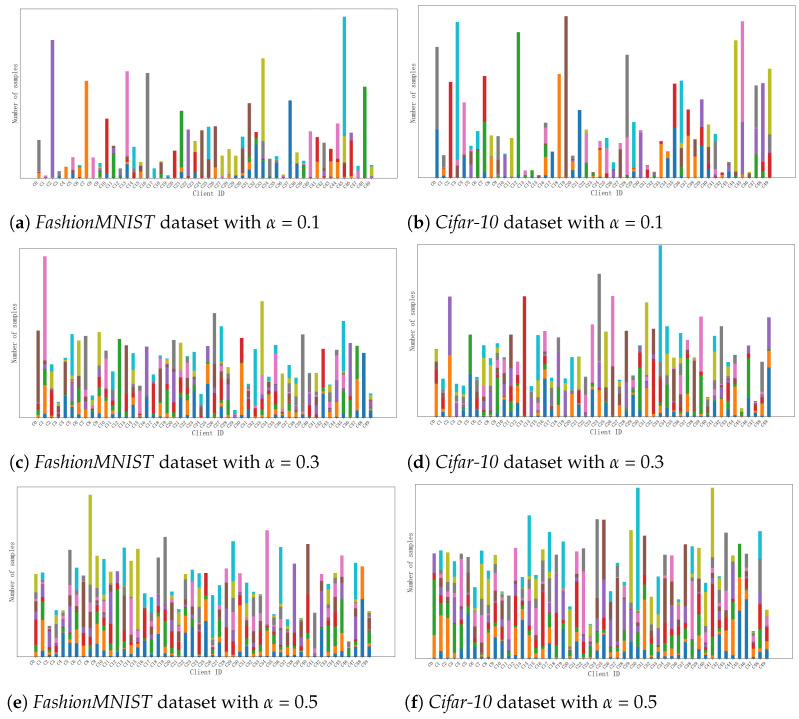
Dirichlet distributions of *FashionMNIST* and *Cifar-10* datasets at three degrees of dispersion.

Within this distribution, the degree of data heterogeneity among different clients is controlled by the parameter α. Adjusting the value of α allows for data partitions with varying degrees of dispersion. When α is larger, the sample data distribution among participants is more similar. Specifically, as α→+∞, the local data distribution of each client becomes consistent with the distribution of the original dataset, and the Dirichlet distribution degenerates into a deterministic distribution at its mean. Conversely, when α is smaller, the distribution differences among local data on each client increase. Particularly, as α→0, each client contains samples from only one randomly selected category. In this paper, to illustrate the varying degrees of personalization among client data, we set the value of α to 0.1, 0.3, and 0.5, thereby simulating Non-IID data distributions under three scenarios. Assuming there are a total of 50 clients in this study, the data partitioning scenarios for these 50 clients under the three distributions are shown in Figure 4.

### 5.2. Experimental Configuration and Evaluation Metrics

This section primarily introduces the preparations conducted before the experiments. The first subsection discusses the configuration of various hyperparameters used in the experiments, while the second subsection outlines all the evaluation metrics involved in the experimental process.

#### 5.2.1. Experimental Configuration

Given the large number of clients typically present in edge computing scenarios and the limited computational capabilities of edge servers to aggregate local models from all clients, we assume a scenario with 50 clients. In each training round, 14% of these clients are selected to participate in the training of the global model. The local learning rate for each client is set to 0.005. The number of global and local iterations is set to 2000 and 1, respectively. Clients’ local data is divided into multiple batches, each containing 64 data points. During the local conditional weighted update, each local model weight κ is set to 0.2. To reflect the data distribution under different levels of dispersion, the Dirichlet coefficient α (represented as dir) is set to 0.1, 0.3, and 0.5 for the dataset partitioning.

#### 5.2.2. Evaluation Metrics

To gain a deeper understanding of the performance of the optimization algorithms, we employed two primary evaluation metrics: the test accuracy of the global model and the value of the loss function. The test accuracy directly reflects the model’s generalization ability on unseen data, while the global model’s loss function reveals the average error across the entire test set, providing us with another perspective to assess the merits of the model’s performance:

(1) Test Accuracy: This is one of the direct indicators of model performance. A high test accuracy implies that the model can accurately predict or classify unseen data, indicating good generalization capabilities of the model.

(2) Loss Function Value: The loss function is a core concept in machine learning, quantifying the discrepancy between the model’s predictions and the actual values. It provides important information about the accuracy of the model’s predictions. A low value of the loss function typically means that the model’s predictions are very close to the true labels, whereas a high value indicates a significant deviation between the model’s predictions and the actual situation.

Furthermore, we compared various metrics such as the maximum, minimum, and average values of all clients’ accuracy and loss function over the last 20 rounds for each algorithm and illustrated the results using box plots. These box plots provide an important perspective on the consistency of the algorithms, offering a clear visual representation of the performance distribution across different clients. Through these diagrams, we can quickly identify fluctuations in algorithm performance and potential outliers. These statistical metrics and visualization tools aid in our deeper understanding of the robustness of the algorithms in a multi-client environment:

(1) Maximum Value: This reflects the highest level of performance that the algorithm can achieve across all clients, which is instrumental in assessing the upper limit of the algorithm’s potential.

(2) Minimum Value: This indicates the lower bound of performance, which is crucial for evaluating the worst-case scenario of the algorithm. Especially in practical applications, it is essential to ensure that the algorithm can meet a certain basic performance standard for all clients.

(3) Average Value: This provides a measure of the algorithm’s average performance across all clients, serving as an important indicator for assessing the overall effectiveness of the algorithm.

(4) Variance or Standard Deviation: This measures the consistency of performance across clients. A lower variance indicates more uniform performance by the algorithm on different clients, which is very important to ensure a good experience for all users.

(5) Quartiles: The quartiles in the box plot, including the median (second quartile), as well as the first (Q1) and third (Q3) quartiles, can show the distribution of the data. These metrics help us understand the central tendency and dispersion of the data.

Through the aforementioned performance metrics, we can comprehensively assess the generalization ability and performance stability of the optimization algorithms across various clients. Moreover, the insights gained from these analyses can guide us in further fine-tuning the details of the algorithms to enhance the minimum performance standards on all clients, while reducing performance variability, thus ensuring the robustness and reliability of the model. Ultimately, we hope that these comprehensive analyses will provide valuable insights and data support for the design and improvement of future optimization algorithms.

### 5.3. Experimental Results

This section presents the experimental results. The first subsection demonstrates, through experiments, the performance decline of the FedAvg algorithm in edge computing scenarios compared to an ideal baseline, highlighting the necessity for algorithm optimization. The second subsection conducts comparative training on each step of our algorithm to validate the effectiveness of each component. The third subsection organizes the algorithms from each step and presents box plots to visualize the performance of all clients across these algorithms. The fourth subsection features a horizontal comparison, contrasting our optimized algorithm with several state-of-the-art approaches to underscore the significance of the proposed method.

#### 5.3.1. Differences between Edge Computing Scenarios and Traditional Federated Learning Scenarios

Traditional federated learning models often presuppose that the data across all clients are independently and identically distributed (*IID*), and that each global model training iteration aggregates the model parameters from all clients, thereby ensuring the effectiveness of the global model on the local data of all clients. However, in edge computing scenarios, the data among different clients are heterogeneous, and due to reasons such as the excessive number of edge-side clients and insufficient computational capabilities at the edge, edge servers are unable to aggregate the local models from all clients, leading to a decline in the accuracy of federated model training. Therefore, we proceed with the following comparisons from the two scenarios (dir = 0.1 for edge scenarios) mentioned above:

(1) A comparison between training with all clients participating and training with a random selection of clients in heterogeneous scenarios.

(2) A comparison between training with a random selection of clients in *IID* data scenarios and heterogeneous data scenarios.

(3) A comparison between traditional *IID* federated learning and heterogeneous federated learning in edge computing scenarios.

**Figure 5:**
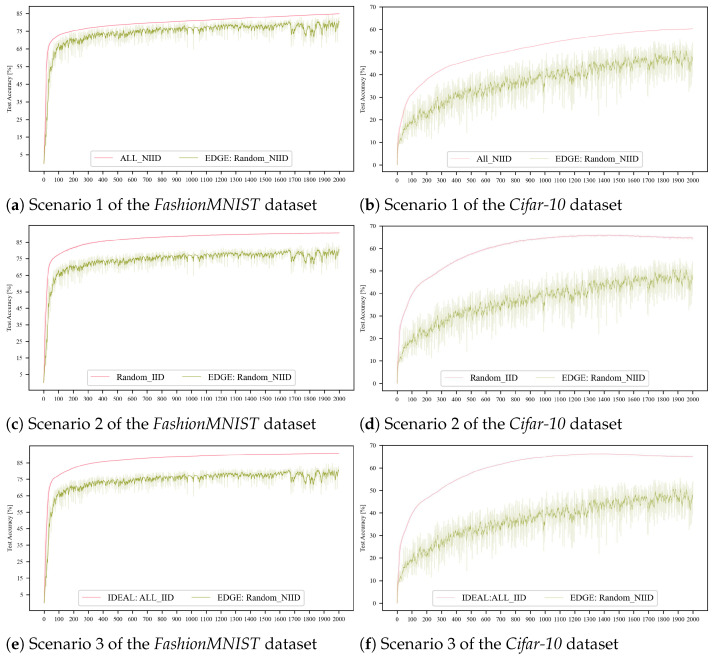
The comparison of model performance on the *FashionMnist* and *Cifar-10* datasets under three edge-case data-distribution scenarios against the ideal situation highlights the importance of client selection in edge scenarios.

These comparisons aim to demonstrate the necessity of optimizing the participation of some clients in the global model training of federated learning in edge computing scenarios, as illustrated in Figure 5.

#### 5.3.2. Verification of the Proposed Optimization Methods for Federated Learning Performance

To enhance the performance of federated learning models in edge computing scenarios, this paper has implemented a series of optimization strategies. Specifically, the optimization efforts mainly involve four key steps: adaptive selection of clients denoted as *F1*, adaptive aggregation of models represented as *F1_F2*, incorporation of cosine similarity during model aggregation indicated as *F1_F2_cos*, and local conditional weight updates at the client-side referred to as *APCSMA*. These strategies are designed to improve the learning efficiency and performance of the model under conditions of non-uniform data distribution. To validate the effectiveness of each step in our algorithm, we have separately recorded the accuracy and variations in the loss function under an ideal scenario (*IDEAL*), a random edge scenario (*FedAvg*), and the four steps outlined in this paper. To reflect the actual conditions of different datasets and edge scenarios, extensive comparative experiments were conducted on the *FashionMnist* and *Cifar-10* datasets across three levels of data-distribution discreteness, corresponding to Dirichlet distributions with dir = 0.1, 0.3, 0.5.

However, to simulate the realistic situation of low client participation rates in edge scenarios, we selected only a small fraction of clients to participate in the global model aggregation each round. This led to fluctuations in the global model performance. Therefore, we employed the Exponential Moving Average (*EMA*) method for curve smoothing in the presentation of our results, with a smoothing factor set to 0.2. This approach allowed us to obtain smoother training progress curves, which more accurately reflect the actual changes in model performance.

**Figure 6:**
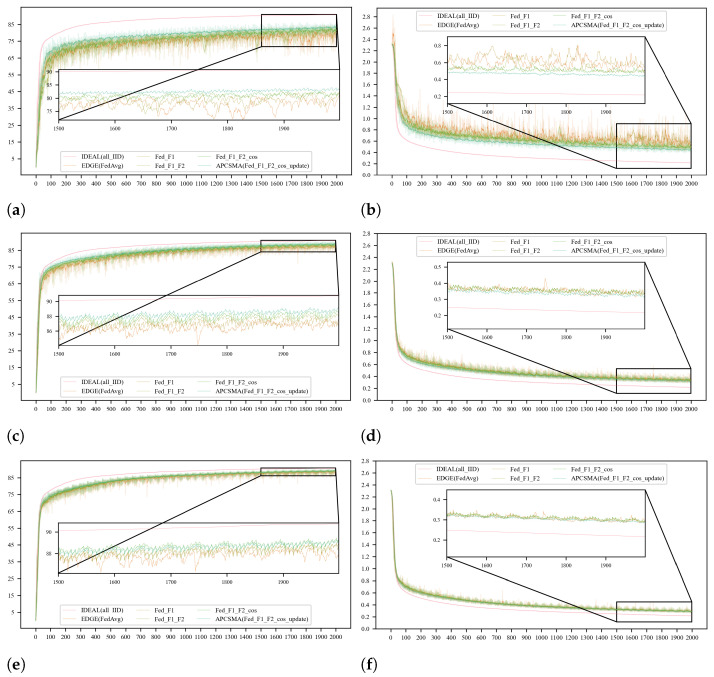
The comparison of the accuracy and loss function values of each algorithm under the three degrees of dispersion of the *FashionMnist* dataset. (**a**) Comparison of accuracy at dir = 0.1. (**b**) Comparison of loss function values at dir = 0.1. (**c**) Comparison of accuracy at dir = 0.3. (**d**) Comparison of loss function values at dir = 0.3. (**e**) Comparison of accuracy at dir = 0.5. (**f**) Comparison of loss function values at dir = 0.5.

Initially, we conducted comparative experiments on the *FashionMnist* dataset and created line charts comparing accuracy and loss function values, as illustrated in Figure 6.

**Figure 7:**
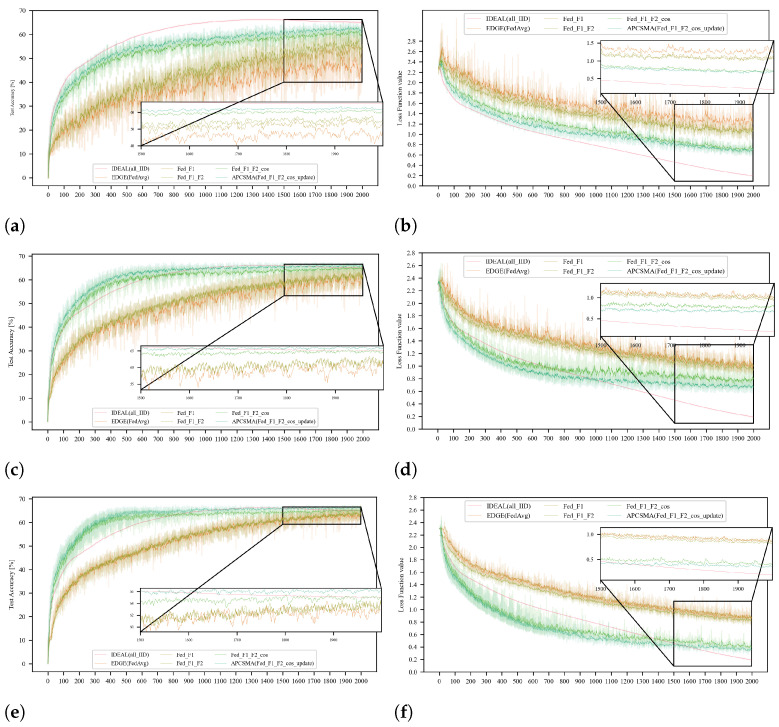
The comparison of the accuracy and loss function values of each algorithm under the three degrees of dispersion of the *Cifar-10* dataset. (**a**) Comparison of accuracy at dir = 0.1. (**b**) Comparison of loss function values at dir = 0.1. (**c**) Comparison of accuracy at dir = 0.3. (**d**) Comparison of loss function values at dir = 0.3. (**e**) Comparison of accuracy at dir = 0.5. (**f**) Comparison of loss function values at dir = 0.5.

Subsequently, to demonstrate the generalizability of our algorithm, we replicated the aforementioned experiments on the *Cifar-10* dataset, as shown in Figure 7. Given that the *Cifar-10* dataset is more complex than the *FashionMnist* dataset, the experimental results are more compelling.

#### 5.3.3. Analysis of Experimental Results

To quantitatively and visually ascertain the percentage improvement offered by the *APCSMA* algorithm across all data distributions, we conducted a step-by-step comparative analysis between the *APCSMA* and *FedAvg* algorithms. Furthermore, we compiled the accuracies of the last 20 rounds for all algorithms under every data-distribution scenario into Table 1. This tabular representation facilitates a more intuitive demonstration of the efficacy of each step within the *APCSMA* algorithm.

From the analysis of the comparative data on accuracy and loss function values for each algorithm under three different data-distribution conditions, as presented in the above figures, a clear trend can be observed: the greater the degree of dispersion in the data distribution among clients, the more significant the impact on the training of the global model, resulting in a notable performance degradation in edge computing scenarios compared to an ideal environment. However, it is noteworthy that, as the dispersion of client data distribution increases, our proposed *APCSMA* algorithm demonstrates a more pronounced effect in optimizing the performance of federated learning models. Specifically, when the data-distribution parameter dir is set to 0.1, 0.3, and 0.5, respectively, the APCMSA algorithm improves the model’s accuracy by 3.9%, 1.9%, and 1.1% on the FashionMnist dataset, and by 31.9%, 8.4%, and 5.4% on the Cifar-10 dataset, respectively.

**Figure 8:**
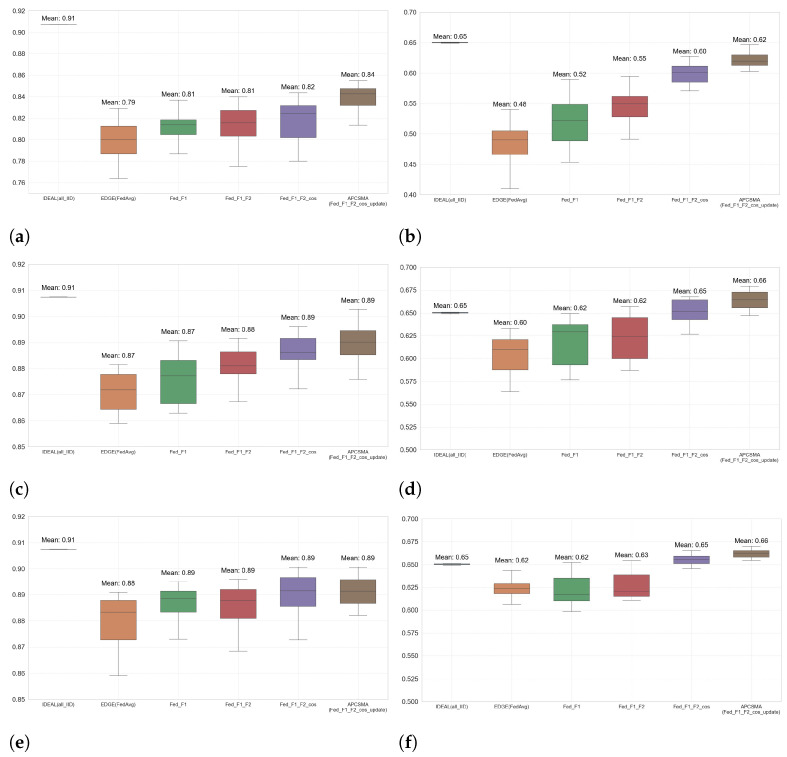
The boxplots for each step of the *APCSMA* algorithm on *FashionMnist* and *Cifar-10* datasets across three levels of discretization. (**a**) Boxplot for the *FashionMnist* Dataset at dir = 0.1. (**b**) Boxplot for the *Cifar-10* Dataset at dir = 0.1. (**c**) Boxplot for the *FashionMnist* Dataset at dir = 0.3. (**d**) Boxplot for the *Cifar-10* Dataset at dir = 0.3. (**e**) Boxplot for the *FashionMnist* Dataset at dir = 0.5. (**f**) Boxplot for the *Cifar-10* Dataset at dir = 0.5.

To delve deeper into the universal applicability of the global model across all clients, this study conducted a detailed analysis of the last 20 rounds of the federated learning process and plotted box plots for the performance of each algorithm across all clients during this period. The maximum and minimum values in these box plots represent the highest and lowest accuracies achieved by the global model on local validations of all clients under specific data-distribution conditions, using the respective algorithms. Furthermore, the width of the box in the box plots reflects the variability in performance across all clients when adopting the algorithm; a narrower box indicates higher fairness of the algorithm across different clients, while a wider box suggests deficiencies in the algorithm’s ability to balance performance across all clients. The specific box plots are displayed in Figure 8.

Through the analysis of these box plots, we have not only confirmed that the *APCSMA* algorithm can effectively enhance the overall performance of the global model but also revealed its potential in reducing the performance disparity among different clients. This finding underscores the superiority of the *APCSMA* algorithm in handling federated learning scenarios with uneven data distribution, providing valuable insights for the optimization of future federated learning models.

### 5.4. Horizontal Comparative Experiment

In our study, to comprehensively evaluate the performance of the proposed methods, we set up comparative experiments involving two representative benchmark algorithms: the *FedPHP* algorithm [5] and the *FedALA* algorithm [6]. The *FedPHP* algorithm takes a moving average of personalized models on each client and uses them to supervise the newly downloaded model in the next global round. The *FedALA* algorithmadaptively aggregates the global model and local models to align with the local objectives, capturing the necessary information from the global model in an element-wise manner. To demonstrate the optimization effects of all algorithms, training performances under ideal conditions (IDEAL) and the random selection algorithm *FedAvg* were also included.

**Figure 9:**
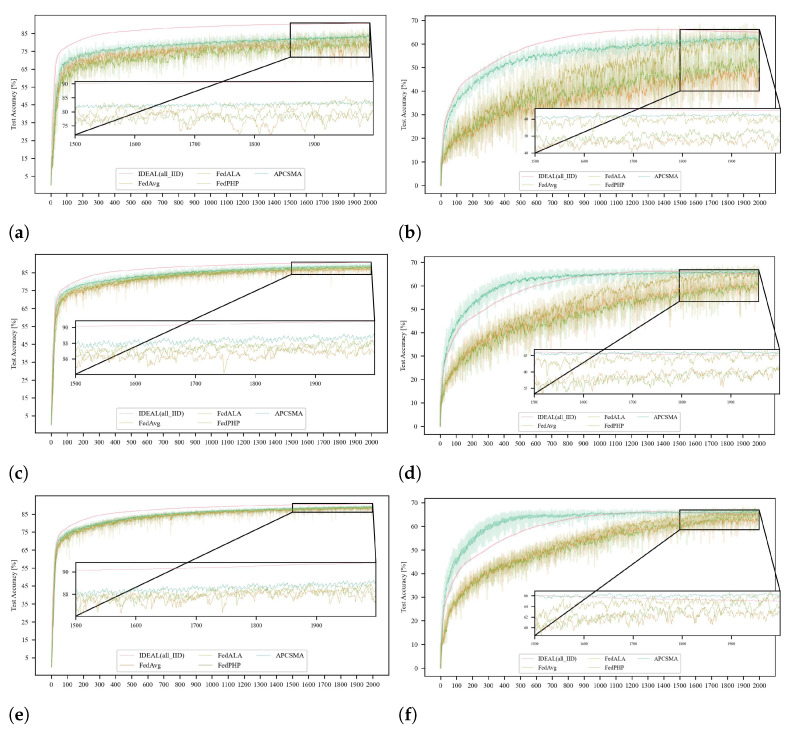
Comparative analysis with *FedPHP* and *FedALA* across three levels of data dispersity on the *FashionMnist* and *Cifar-10* Datasets. (**a**) Horizontal Comparison at a Dispersity Level of dir = 0.1 on the *FashionMnist* Dataset. (**b**) Horizontal Comparison at a Dispersity Level of dir = 0.1 on the *Cifar-10* Dataset. (**c**) Horizontal Comparison at a Dispersity Level of dir = 0.3 on the *FashionMnist* Dataset. (**d**) Horizontal Comparison at a Dispersity Level of dir = 0.3 on the *Cifar-10* Dataset. (**e**) Horizontal Comparison at a Dispersity Level of dir = 0.5 on the *FashionMnist* Dataset. (**f**) Horizontal Comparison at a Dispersity Level of dir = 0.5 on the *Cifar-10* Dataset.

In the comparative experiments, we also trained on three levels of data heterogeneity within the *FashionMnist* and *Cifar-10* datasets. The line graphs and loss function plots were smoothed using an exponential moving average with a smoothing factor of 0.2, and the final accuracy and loss function comparison charts are shown in Figure 9.

To present the results of the horizontal comparison more intuitively, we summarized the accuracy of different algorithms under various scenarios in Table 2, finding that our algorithm surpasses the existing algorithms *FedAvg*, *FedPHP*, and *FedALA*.

## 6. Conclusions

In this study, we have proposed an innovative solution for federated learning problems in edge computing scenarios: the *APCSMA* algorithm. This algorithm designs a contribution function during the client-selection and model-aggregation phases, integrating a cosine similarity metric within the contribution function at the model-aggregation stage to enhance the personalized learning capabilities of the model. Additionally, we use a conditional weighted update strategy to optimize the local model update process, aiming to further improve model performance.

To validate the effectiveness of the proposed algorithm, we conducted a series of experiments on the *FashionMnist* and *Cifar-10* datasets, evaluating the algorithm when the data-distribution parameter dir is set to 0.1, 0.3, and 0.5, respectively. The experimental results demonstrate that, compared to existing federated learning algorithms, the *APCSMA* algorithm achieved significant improvements in accuracy: 3.9%, 1.9%, and 1.1% on the *FashionMnist* dataset, and 31.9%, 8.4%, and 5.4% on the *Cifar-10* dataset, respectively. These results not only prove the *APCSMA* algorithm’s effectiveness in handling *Non-IID* data issues in edge computing scenarios but also indicate that the more complex and dispersed the client data, the more significant the improvement of our algorithm. Furthermore, we conducted a horizontal comparison with two current advanced algorithms, *FedPHP* and *FedALA*, thereby demonstrating the performance optimization and enhancement of our algorithm.

In conclusion, the *APCSMA* algorithm provides a novel and effective solution for solving the federated learning problem in edge computing scenarios. We expect that this study will bring a new perspective to academic research and practice in this area and provide insights for future research.

At the same time, we are clearly aware of the limitations of the current study. In this paper, we have compared the *APCSMA* algorithm with *FedAvg*, *FedPHP*, and *FedALA*; however, there are numerous other research papers in this area. In our future work, we will conduct a broader range of comparative experiments to further explore the advantages, contributions, and potential limitations of *APCSMA*, as well as propose more creative solutions.

We will continue to optimize the contribution function, comprehensively consider “edge” characteristics, and investigate the effectiveness of APCSMA across more diverse datasets and more complex application scenarios. Additionally, we will consider the integration of APCSMA with Federated Multi-Task Learning methods [35] to effectively address data privacy and security issues, thereby further enhancing the model’s performance and generalization capabilities.

## Figures and Tables

**Table 1:** Summary of optimization results.

		FashinMnist			Cifar-10	
Algorithm	dir = 0.1	dir = 0.3	dir = 0.5	dir = 0.1	dir = 0.3	dir = 0.5
Ideal	0.907	0.907	0.907	0.650	0.650	0.650
FedAvg	0.795	0.870	0.880	0.470	0.608	0.627
F1	0.809	0.873	0.886	0.521	0.612	0.631
F1+F2	0.814	0.881	0.886	0.548	0.613	0.636
F1+cos F2	0.817	0.885	0.890	0.600	0.645	0.650
APCSMA	0.834	0.889	0.891	0.620	0.659	0.661

**Table 2:** Summary of optimization results.

		FashinMnist			Cifar-10	
Algorithm	dir = 0.1	dir = 0.3	dir = 0.5	dir = 0.1	dir = 0.3	dir = 0.5
Ideal	0.907	0.907	0.907	0.650	0.650	0.650
FedAvg	0.795	0.870	0.880	0.470	0.608	0.627
FedPHP	0.793	0.879	0.878	0.494	0.600	0.635
FedALA	0.832	0.883	0.887	0.603	0.658	0.657
APCSMA	0.834	0.889	0.891	0.620	0.659	0.661

## Data Availability

The data that support the findings of this study are available from the corresponding author upon reasonable request.
